# Prognostic significance of circulating tumor DNA alterations in advanced renal cell carcinoma from SCRUM-Japan MONSTAR-SCREEN: a nationwide genomic profiling project

**DOI:** 10.1038/s41416-025-02985-8

**Published:** 2025-05-05

**Authors:** Taigo Kato, Masaki Shiota, Koshiro Nishimoto, Nobuaki Matsubara, Takahiro Osawa, Takashige Abe, Yota Yasumizu, Nobuyuki Tanaka, Yoshiyuki Yamamoto, Yu Ishizuya, Hikaru Abutani, Hideaki Bando, Takao Fujisawa, Yoshiaki Nakamura, Mototsugu Oya, Nobuo Shinohara, Masatoshi Eto, Takayuki Yoshino, Norio Nonomura

**Affiliations:** 1 Department of Urology, Osaka University Graduate School of Medicine, Osaka, Japan; 2 Department of Urology, Kyushu University Graduate School of Medical Science, Fukuoka, Japan; 3 Department of Uro-Oncology, Saitama Medical University International Medical Center, Saitama, Japan; 4 Department of Urology, Faculty of Medicine, Miyazaki University Hospital, Miyazaki, Japan; 5 Department of Medical Oncology, National Cancer Center Hospital East, Kashiwa, Japan; 6 Department of Renal and Genitourinary Surgery, Hokkaido University Graduate School of Medicine, Sapporo, Japan; 7 Department of Urology, Keio University School of Medicine, Tokyo, Japan; 8 Chugai Pharmaceutical Co., Ltd, Tokyo, Japan; 9 Department of Gastroenterology and Gastrointestinal Oncology, National Cancer Center Hospital East, Kashiwa, Japan; 10 Translational Research Support Office, National Cancer Center Hospital East, Kashiwa, Japan; 11 Department of Head and Neck Medical Oncology, National Cancer Center Hospital East, Kashiwa, Japan; 12 Department of International Research Promotion Office, National Cancer Center Hospital East, Kashiwa, Japan; 13 Department of the Promotion of Drug & Diagnostic Development, National Cancer Center Hospital East, Kashiwa, Japan

**Keywords:** Renal cell carcinoma, Cancer genomics

## Abstract

**Background:**

Circulating tumor DNA (ctDNA) is a promising tool for diagnosing and predicting cancer prognosis. However, its clinical utility in metastatic renal cell carcinoma (mRCC) remains unclear, particularly in terms of clinical prognosis.

**Methods:**

We enrolled 124 patients with mRCC in the MONSTAR-SCREEN study (UMIN 000036749) between August 2019 and February 2022, a national observational ctDNA-based screening study, and performed ctDNA sequencing before and at the time of resistance to systemic therapy.

**Results:**

ctDNA were assessed in 178 samples containing 432 mutations. The most frequently altered genes at baseline were *VHL* (25.0%), *PBRM1* (10.9%), *TERT2* (8.7%), *BAP1* (8.7%), and *MTOR* (7.6%). Patients receiving first-line therapy with tumor fraction (TF) < 1.2% showed significantly better progression-free survival than those with TF ≥ 1.2% (Hazard ratio (HR) = 0.467; 95% CI 0.229–0.979; *p *= 0.0425). *BAP1* mutational status of ctDNA at baseline led to poor OS (HR = 0.4867; 95% CI 0.322–0.736; *p *= 0.0003). Serial ctDNA analysis showed that 46.8% of patients developed new ctDNA mutations at disease progression, which was linked to shorter time to progression (*p *= 0.046).

**Conclusions:**

Our findings demonstrated that ctDNA profiling is feasible in mRCC and can predict disease progression after treatment.

## Introduction

Vascular endothelial growth factor (VEGF)-targeted therapies and the emergence of immune checkpoint inhibitors (ICIs) have substantially improved the clinical outcomes of patients with metastatic renal cell carcinoma (mRCC) [1, 2], specifically transforming it from a rapidly progressing fatal disease through several subsequent therapies.

However, a reliable marker for predicting response or resistance to these treatments is lacking, posing challenges to precision oncology. We initiated MONSTAR-SCREEN, a nationwide solid cancer biomarker screening project within the SCRUM-Japan network, to assess chronological tumor evolution and intratumoral genomic heterogeneity for accurate treatment selection with the aim to accelerate innovation in therapies [3–6]. In particular, we focused on circulating tumor DNAs (ctDNAs) as an alternative to tissue genotyping owing to their ability to detect genomic alterations with high accuracy across solid tumors [7]. In this project, we attempted to characterize the DNA genomic profile of cancers presenting with lower ctDNA levels, including RCC, head and neck cancer, gynecological cancer, and malignant melanoma, in a large cohort [3, 5].

In the present study, we analyzed a national cohort of patients with mRCC who underwent serial liquid biopsies, using a clinical ctDNA assay to characterize ctDNA changes over time. We linked the mutational profiles to clinical data, including prognosis and treatment, for the overall cohort.

## Materials and methods

### Patient enrollment

SCRUM-Japan MONSTAR-SCREEN was a nationwide cancer genome screening project that prospectively evaluated ctDNA in patients with advanced solid tumors, except lung cancer, across 31 Japanese institutions (UMIN 000036749). The analysis was conducted on patients who had been registered with MONSTAR-SCREEN between August 2019 and February 2022 with a median follow-up of 20.2 months (range 3.5–36.5). The primary eligibility criteria were the presence of histologically proven solid tumors, unresectable lesions, and an Eastern Cooperative Oncology Group performance status of 0–1. The study protocol was approved by the Institutional Review Board of each participating institution; all patients provided written informed consent.

Tissue samples were collected before treatment initiation, whereas blood samples were collected before treatment initiation and during disease progression. Blood samples were profiled using FoundationOne®Liquid CDx (F1LCDx®, Foundation Medicine, Cambridge, MA, USA). Tissue samples were profiled using FoundationOne®CDx (F1CDx®, Foundation Medicine).

### Comprehensive genomic profiling of ctDNA

All comprehensive genomic profiling assays were conducted in a laboratory certified under the Clinical Laboratory Improvement Amendments (CLIA), accredited by the College of American Pathologists (CAP) and approved by the New York State (Foundation Medicine, Inc., Cambridge, MA, USA). Circulating cell-free DNA (cfDNA) was extracted from whole-blood samples and analyzed using F1LCDx, a validated in vitro diagnostic device targeting 324 cancer-related genes. This assay employs hybrid-capture technology and deep sequencing coverage to detect single-nucleotide variants, indels, genomic rearrangements, copy number amplifications and losses, as well as genomic signatures such as bTMB, MSI, and tumor fraction (TF), which estimate the proportion of ctDNA in the cfDNA extracted from plasma. The Foundation Medicine’s ctDNA TF on F1LCDx® uses a composite algorithm that prioritizes aneuploidy at higher levels to minimize germline signals and prioritizes variant allele frequencies of canonical alterations at lower levels to maximize the dynamic range, merging two methods for ctDNA TF estimation. When the absence of detectable tumor aneuploidy restricts the estimation of TF, a variant-based approach is employed. This involves identifying the nongermline variant, excluding specific clonal hematopoiesis (CH)-associated alterations, as described previously [8, 9].

To examine changes in ctDNA over time, alterations were classified as emergent (not detectable at baseline but detectable at any variant allele frequency (VAF) during progression), increasing (detectable at baseline with increase in VAF by 20% or greater during progression), stable (detectable at baseline with less than 20% increase or decrease in VAF during progression), decreasing (detectable at baseline with decline in VAF by 20% or greater during progression), or lost (detectable at baseline at any VAF but not detectable at progression) phases.

### Clinical data

Response assessment eligibility was based on at least one scan following treatment initiation or clinical progression after treatment initiation. Tumor assessments were performed using the Response Evaluation Criteria in Solid Tumors, version 1.1, at screening and every 2–3 months from the start of treatment. Time to progression was defined as the time from the start of treatment to clinical and/or radiographic progression or death from any cause.

### Statistical analysis

Differences between the two groups and maximal VAF at baseline in patients with emergent and other types of mutations were compared using Fisher’s exact tests for categorical variables. Comparisons of mean time to progression were performed using Mann–Whitney test. Progression-free survival (PFS) and overall survival (OS) were estimated using the Kaplan–Meier method and compared using log-rank tests. Differences were considered statistically significant at *p* < 0.05. All statistical analyses were conducted using JMP (version 15.0; SAS Institute, Cary, NC, USA).

## Results

### Patient characteristics

In total, 124 patients with mRCC were enrolled in the MONSTAR-Urology group; 110 patients were analyzed after excluding those whose clinical data were unavailable (*n* = 3) and those with undetected ctDNA (*n* = 11) (Table 1, Supplementary Fig. S1, Supplementary Table S1). The median patient age was 66 years (range, 21–83). The most prevalent histological type was clear cell carcinoma, present in 110 (91.0%) patients. The number of metastatic sites ranged from one to five; 66 (54.5%) patients had multiple metastases at baseline. The most common sites of distant metastasis were the lungs (68.6%), followed by the lymph nodes (38.8%) and bones (25.6%). Specifically, 61.2% and 21.5% of patients were classified as intermediate and poor risk, respectively, according to the IMDC risk classification. Nephrectomies and tissue biopsy had previously been conducted in 74 (61.2%) and 33 (27.3%) patients, respectively.

**Table 1:** Characteristics of the entire cohort.

Characteristics	
Median age, years (range)	66 (21–83)
Sex, *n* (%)	
Male	92 (76.0)
Female	29 (24.0)
Histopathology, *n* (%)	
Clear cell RCC	110 (91.0)
Non-clear cell RCC	11 (9.0)
IMDC risk, *n* (%)	
Favorable	21 (17.4)
Intermediate	74 (61.2)
Poor	26 (21.5)
Number of metastatic organs, *n* (%)	
Single	55 (45.5)
Multiple	66 (54.5)
Metastatic site	
Bone	31 (25.6)
Lung	83 (68.6)
Liver	17 (14.0)
Lymph nodes	47 (38.8)
Brain	4 (3.3)
Confirmation of tissue type, *n* (%)	
Nephrectomy	74 (61.2)
Biopsy	33 (27.3)
Unknown	4 (3.3)
Treatment line, *n* (%)	
1st	90 (74.4)
2nd	15 (12.4)
3rd	6 (6.7)
≥4th	10 (11.1)
CRP, *n* (%)	
Low (<1.0)	71 (58.7)
High (≥1.0)	43 (35.5)
Unknown	7 (5.8)

*CRP* C-reactive protein, *IMDC* International Metastatic RCC Database Consortium, *RCC* renal cell carcinoma.

**Fig. 1:**
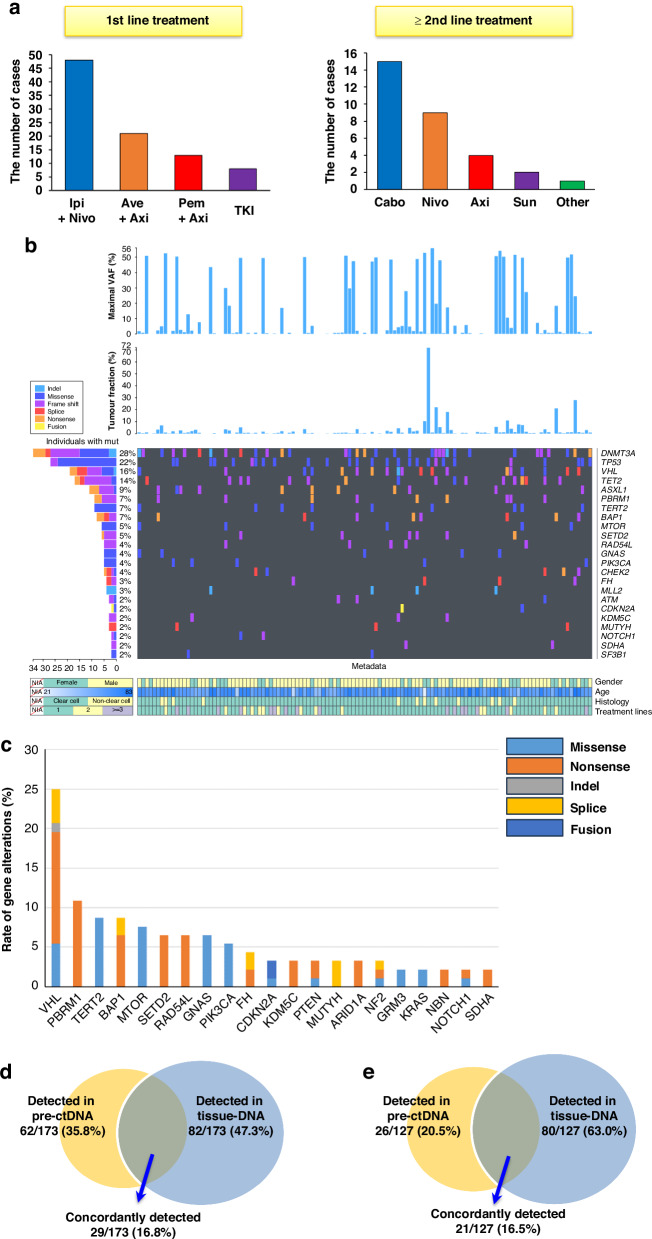
Genomic alterations in circulating tumor DNA before initiating treatments. **a** Distribution of first-line and post-first-line therapies. Ipi+Nivo Ipilimumab plus nivolumab, Ave+Axi Avelumab plus axitinib, Pem+Axi Pembrolizumab plus axitinib, TKI Tyrosine kinase inhibitor, Cabo Cabozantinib, Nivo Nivolumab, Axi Axitinib, Sun Sunitinib. **b** Circulating tumor DNA (ctDNA) genomic landscape in metastatic renal cell carcinoma at treatment baseline. Genomic alterations observed more than 2% of all cases were listed. **c** Cumulative genomic alterations in ctDNA at baseline after the exclusion of genes associated with clonal hematopoiesis of indeterminate potential (CHIP). **d** The frequency of overlapping and exclusive genomic alterations in ctDNA and tissue-based profiling in the overall cohort. **e** The frequency of overlapping and exclusive genomic alterations in ctDNA and tissue-based profiling after exclusion of genes associated with CHIP.

During the observational period, 90 patients (74.4%) were enrolled for first-line treatment, whereas 31 patients (25.6%) received more than one treatment as prior therapy Supplementary Table S1). Notably, 90% of patients who received first-line therapy received ICI combination therapy, reflecting the current trends in mRCC treatment (Fig. 1a).

### ctDNA assessment at treatment baseline

Of all samples at baseline, ctDNA genomic landscape with a mutation rate of more than 2% were demonstrated according to maximal VAF and TF (Fig. 1b). Overall, at least one ctDNA pathogenic genomic alteration (GA) was identified in 91 samples (82.7%), with a median of three GA per patient (interquartile range, 2–4 GAs; Supplementary Fig. S2). The most common pathogenic GAs were *DNMT3A* (28%), *TP53* (22%), *VHL* (16%), *TET2* (14%), and *ASXL1* (9%) (Fig. 1b, Supplementary Fig. S2). The median value of maximal VAF was 4.88% (interquartile range, 0.73–47.18%). After exclusion of genes univocally associated with clonal hematopoiesis of indeterminate potential (CHIP; *ATM*, *ASXL1*, *CBL*, *CHEK2*, *DNMT3A*, *JAK2*, *IDH2*, *KMT2D (MLL2)*, *MPL*, *MYD88*, *SF3B1*, *TET2*, *TP53*, and *U2AF1*), the five most frequent cancer-related alterations identified through ctDNA sequencing were *VHL* (25.0%), *PBRM1* (10.9%), *TERT2* (8.7%), *BAP1* (8.7%), and *MTOR* (7.6%) mutations (Fig. 1c).

Next, we explored the concordance of mutational profiles between ctDNA and tumor DNA (Fig. 1d). Of the total 173 mutations, 29 mutations (16.8%) were consistent with somatic mutations detected in tumor DNA, whereas 62 mutations were identified only in ctDNA. The concordance rate between cfDNA and tumor DNA was 16.5% (21 of 127 mutations) after excluding genes associated with CHIP (Fig. 1e), suggesting that cfDNA identifies possible relevant GAs that are not captured by single-lesion tumor biopsy.

### Clinical prognosis depending on baseline ctDNA status

We investigated the TF in ctDNA in all patients at baseline to evaluate whether ctDNA information could facilitate clinical benefit prediction. The median TF was 1.15% (interquartile range, 0.62–2.85%) in this cohort (Supplementary Fig. S3). We analyzed the concordance between bTMB and TMB in our cohort. Contrary to expectations, there was no significant correlation between the two parameters, even in samples with TF > 1% (Supplementary Fig. S4).

**Fig. 2:**
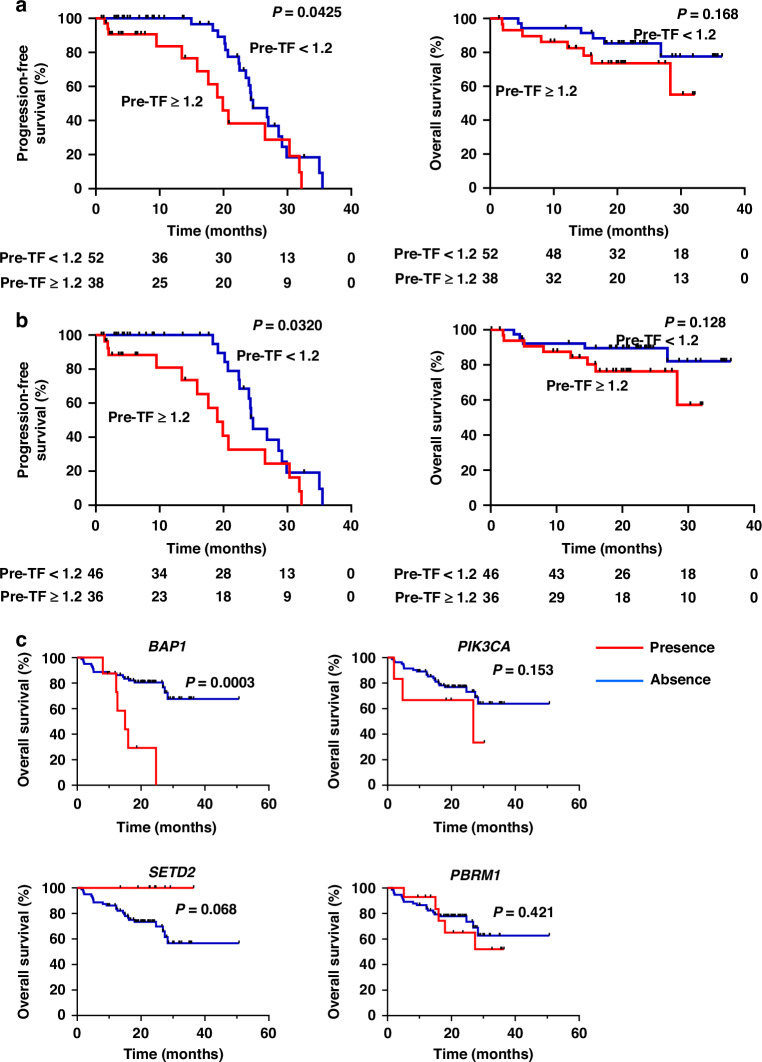
Relationship between baseline tumor fraction of circulating tumor DNA and clinical prognosis. **a** Progression-free survival (PFS) and overall survival (OS) after initiating treatments according to tumor fraction at baseline in the overall cohort. **b** PFS and OS after initiating the combination therapy of first-line immune checkpoint inhibitors according to tumor fraction at baseline. **c** OS depending on the specific mutational status of circulating tumor DNA at baseline. Differences between the two groups were assessed using the log-rank test.

Following the stratification of patients of first-line treatment into two groups, patients with TF < 1.2% showed significantly better PFS than those with TF ≥ 1.2% (median PFS [mPFS], 24.30 versus 19.03 months, hazard ratio [HR] = 0.467, 95% CI; 0.229–0.979, *p* = 0.0425, Fig. 2a). Patients with TF < 1.2% showed a tendency of better OS that those of TF ≥ 1.2% (12-month OS, 91.3% versus 82.4%, HR = 0.469, 95% CI: 0.160–1.379, *p* = 0.168; Fig. 2a). Among patients who received ICI combination therapy, accounting for 92.7% of first-line treatment, patients with TF < 1.2% showed better PFS (mPFS, 24.63 versus 19.03 months, HR = 0.140, 95% CI: 0.632–0.926, *p* = 0.0320, Fig. 2b). Moreover. patients with TF < 1.2% also showed a tendency of better OS that those of TF ≥ 1.2% (12-month OS, 89.5% versus 84.1%, HR = 0.422, 95% CI: 0.139–1.280, *p* = 0.128; Fig. 2b). Notably, in multivariate analysis, TF ≥ 1.2% was significantly associated with reduced PFS in patients with first-line treatment (HR = 1.699, 95% CI: 1.818–3.680, *p* = 0.043, Table 2), whereas IMDC intermediate/poor risk groups (77.7% of all patients) showed a tendency of reduced OS (HR = 1.389, 95% CI: 0.893–3.911, *p* = 0.114). These results suggest that ctDNA fraction status may influence the probability of responses to systemic therapies.

**Table 2:** Univariate and multivariate analyses of PFS and OS using Cox regression models in patients with first-line treatments.

	PFS				OS			
Variable	Univariate analysis		Multivariate analysis		Univariate analysis		Multivariate analysis	
	HR	*p* value	HR	*p* value	HR	*p* value	HR	*p* value
Sex								
Male	Reference							
Female	1.027 (0.435–2.381)	0.749	1.131 (0.577-2.217)	0.720	1.945 (0.850–4.453)	0.128	1.622 (0.668–3.941)	0.284
Age, years								
<75	Reference							
≥75	1.576 (0.149–2.683)	0.596	1.534 (0.142–2.961)	0.577	1.778 (0.605–5.228)	0.296	1.481 (0.538–4.948)	0.385
Number of metastatic sites								
Single	Reference							
Multiple	1.151 (0.587–2.254)	0.683	1.006 (0.451–2.244)	0.988	1.095 (0.637–1.883)	0.742	1.259 (0.552–2.878)	0.417
IMDC risk								
Favorable	Reference							
Intermediate/Poor	1.784 (0.739–4.364)	0.204	2.378 (0.774–8.395)	0.468	2.165 (0.922–5.084)	0.076	1.389 (0.893–3.911)	0.114
CRP								
<1.0	Reference							
≥1.0	1.773 (0.812–4.408)	0.118	2.235 (0.803–3.215)	0.123	1.384 (0.775–2.471)	0.272	1.020 (0.508–2.034)	0.860
Pre-TF								
<1.2%	Reference							
≥1.2%	2.074 (1.174–3.664)	0.014	1.699 (1.818–3.680)	0.043	2.186 (0.976–4.899)	0.057	1.750 (0.976–4.523)	0.148

*HR* hazard ratio, *IMDC* International Metastatic RCC Database Consortium, *TF* tumor fraction.

Moreover, when we examined OS depending on the mutational status of several genes associated with unfavorable prognosis by tissue-based information [10, 11], alterations in *BAP1* in ctDNA appeared to be associated with poor prognosis in patients with mRCC　(mOS, 15.0 versus not reached, HR  =  18.88, 95% CI, 3.787–94.13, *p* = 0.0003, Fig. 2c), implying that ctDNA mutational profiling as an alternative of tissue-based genotyping could predict clinical prognosis in patients with mRCC. On the other hand, we found that *BAP1*, *PIK3CA*, *SETD2*, and *PBRM1* alterations did not affect OS in this cohort (Supplementary Fig. S5).

### Treatment-resistant gene alterations inferred from ctDNA dynamics

**Fig. 3:**
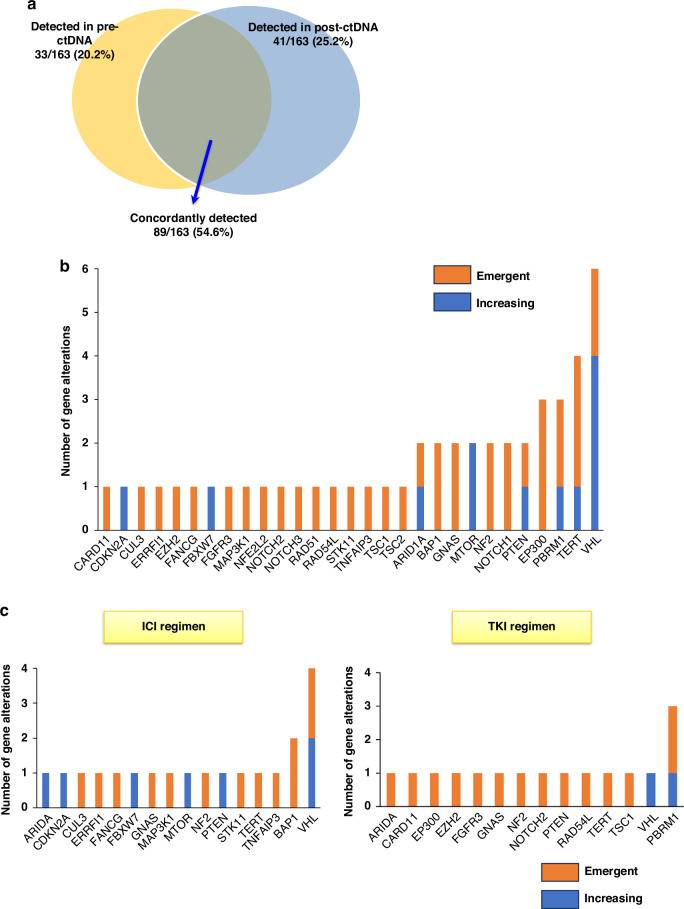
Dynamics of circulating tumor DNA alterations in metastatic renal cell carcinoma. **a** The frequency of overlapping and exclusive genomic alterations in circulating tumor DNA (ctDNA) at baseline and disease progression. **b** Distribution of increasing or emergent gene alterations in ctDNA during disease progression in the overall cohort. **c** Distribution of increasing or emergent gene alterations in ctDNA during disease progression in patients treated with immune checkpoint inhibitors or tyrosine kinase inhibitors.

A proportion of patients showed ctDNA alterations determined through liquid biopsies at baseline and during progression, including 46 patients in our cohort. Among the mutations identified in ctDNA before and after treatment, 54.6% were identified in both, whereas 25.2% were identified only in post-treatment samples (Fig. 3a).

The dynamics of ctDNA can be used to deduce changes linked to treatment resistance, as changes in the detection and VAF of mutations can serve as indicators of pharmacodynamic responses. To delineate the changes in ctDNA alterations during progression, ctDNA alterations were categorized according to their dynamics as emergent, increasing, stable, decreasing, or lost. Among all patients, 49 and 25 unique GAs, including missense, in-frame, truncating, splice site, and copy number alterations in 27 patients, appeared as emergent and increasing GAs in post-treatment ctDNA samples, respectively (Fig. 3b, Supplementary Table S2). Gene-level analysis revealed that mutations in *VHL*, *TERT*, and *PBRM1* were the most common emergent and increasing GAs in our cohort (Fig. 3b).

We subsequently stratified patients into ICI and TKI regimens. To clearly compare the ICI and TKI regimens, we defined patients with nivolumab or nivolumab plus ipilimumab as ICI regimen group, and those with TKI monotherapy as TKI regimen group. The most prevalent emergent and increasing GAs were *VHL* and *BAP1* in the ICI regimen group and *PBRM1* in the TKI regimen group (Fig. 3c).

### Emergent ctDNA alterations affect clinical prognosis

**Fig. 4:**
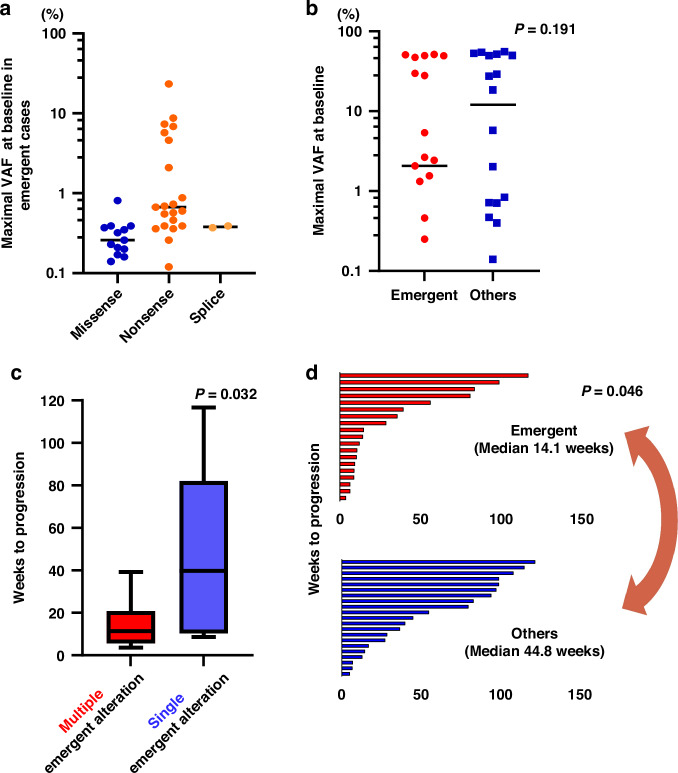
Characteristics of emergent alterations in circulating tumor DNA in metastatic renal cell carcinoma. **a** Maximal variant allele frequency (VAF) of emergent circulating tumor DNA (ctDNA) alterations by mutation type are shown in the dot plot. **b** Maximal VAF of emergent ctDNA alterations in emergent genomic alterations and other types (increasing, stable, decreasing, or lost) of genomic alterations. **c** Mean time to progression was significantly shorter among patients with multiple emergent alterations (polyclonal) versus that in those with single emergent alterations (monoclonal). **d** Mean time to progression was significantly shorter among patients with emergent alterations than that in those with other types of mutations (increasing, stable, and lost). Comparisons of mean time to progression were performed using Mann–Whitney test.

We characterized the clinical features of emergent ctDNA alterations in a national cohort because these mutations may define new therapeutic targets for individual patients. All emergent alterations had a maximal VAF of >0.1%, with a limit of detection of VAF at 0.1% in the liquid biopsy assay (Fig. 4a). There was no significant difference in maximal VAF at baseline between the emergent and other types of GAs (Fig. 4b), suggesting that the baseline maximal VAF was not predictive of emergent alterations in mRCC.

Contrastingly, patients with multiple emergent alterations, likely denoting polyclonal resistance mechanisms, had a shorter time to progression (median 11.5 weeks, range, 3.6–39.2 weeks) than patients with a single emergent alteration (median 39.7 weeks, range, 8.7–116.7 weeks) (*p* = 0.032; Fig. 4c). The mean duration until progression was 14.1 and 44.8 weeks among cases with and without emergent ctDNA alterations, respectively, differing significantly (*p* = 0.046; Fig. 4d). These findings indicate that a high prevalence of emergent ctDNA alterations impairs clinical outcomes during treatment.

Finally, emergent alterations were categorized according to their potential for actionability based on the classification schemes in OncoKB database. Seven emergent alterations (35.0%) were classified as level 1. They were considered clinically actionable using approved or investigational drugs (Supplementary Fig. S6). Level 2 and 3 alterations accounted for 10.0% of patients with emergent alterations. These results indicate that patients with emergent alterations can achieve eligibility criteria for clinical trials.

## Discussion

This study evaluated the prognostic significance of ctDNA alterations in advanced RCC using data from the SCRUM-Japan MONSTAR-SCREEN study. We found that ctDNA mutations were significantly associated with poorer progression-free and overall survival, confirming the study’s aim to establish ctDNA as a valuable prognostic biomarker. Our findings highlight the novelty of ctDNA as a less invasive alternative for monitoring advanced RCC and underscore its potential to enhance personalized treatment strategies.

ctDNA detection, or liquid biopsy, is a non-invasive method of detecting tumor-associated molecular alterations in various cancers [12–14]. However, in a subset of cancers including RCC, pancreatic cancer, and glioma, the effectiveness of ctDNA analysis remains unclear because of the low rate of ctDNA shedding, not allowing for universal usage for cancer screening and prognostication [15–17]. However, recent progress in next-generation sequencing has improved the precision of ctDNA analysis, underscoring its potential value for RCC treatment in clinical practice [18–21]. In this study, we analyzed a large national cohort of patients with mRCC who underwent serial liquid biopsies using a clinical ctDNA assay to elucidate the relationship with prognostication and characterize ctDNA changes over time leading to resistance to systemic therapies.

We demonstrated several novel findings indicating the clinical utility of ctDNA in mRCC. ctDNA analysis revealed a high detection rate of ctDNA in 84.5% of patients with “metastatic” RCC. Our findings partially aligned with those of Pal et al., who described the presence of genomic alterations in 78.6% of patients with mRCC using different cancer gene panels [18]. Several previous studies reported ctDNA detection rates of 17–54% in all RCCs [22–24], which may be due to differences in tumor stages (including localized tumor or not), number of target genes, and sequence depth [25]. There are different predominant mechanisms of cell death in RCC metastases compared with those in prostate or bladder metastases (i.e., ferroptosis, necrosis, or autophagy vs. apoptosis), implying that less fragmented DNA from tumor cells can leak into the blood [26]. Whole-genome sequencing approach with tumor-specific variants (tumor-informed ctDNA analysis) has increased the sensitivity of detection limit of 0.007–0.034% VAF in patients with ICIs and may increase the chance to predict drug responsiveness or resistance in patients with mRCC receiving systemic therapy [27–29]. These methods may further enhance the ctDNA detection and identify clinically relevant GAs. Notably, even with the high detection rate of ctDNA in our cohort, the concordance between ctDNA and tissue-based profiling was limited to 16.8% of all detected variants, suggesting that many baseline ctDNA alterations were not detected by tissue-based DNA sequencing and ctDNA may better capture the heterogeneity of multiple metastases [7].

The high amount of TF at baseline led to poor prognosis in patients with RCC in “metastatic” settings (Fig. 2a). Plasma ctDNA TF is an independent prognostic biomarker in four major advanced tumors [9]. Our results partially aligned with previous data showing that ctDNA-positive patients with mRCC had worse PFS and OS than those without ctDNA [30–32]. Even in the subgroup analysis of patients who received first-line ICI combination therapy, PFS and OS were shorter in patients with high TF levels (Fig. 2b). *BAP1* mutational status of ctDNA at baseline led to poor prognosis (Fig. 2c). These findings suggest that the evaluation of ctDNA before treatment is emerging as a potential alternative to tissue DNA for predicting disease progression with low ctDNA shedding.

Dynamic ctDNA analysis revealed the critical association of emergent alterations with disease progression, affecting patient prognoses. Emergent ctDNA alterations likely originate from subclonal populations that survive treatment-mediated selection and undergo sufficient expansion until ctDNA can be detected. Some specific alterations in *TP53*, *CCNE1*, *GNAS*, and *PIK3CA* are associated with chemotherapy resistance in hepatic cholangiocarcinoma, pancreatic cancer, and esophageal cancer [33]. Another important finding is that a subset of patients with emergent ctDNA alterations may show therapeutic relevance for matched therapies. Hsiehchen et al. reported that 14–27% of patients across histology are eligible for additional clinical trials in the same state of residence [33]. Thus, serial liquid biopsies may be clinically meaningful in a substantial subset of patients even with mRCC, and their value could increase with the growth of biomarker-directed therapies. These results indicate that the abundance of pathogenic emergent ctDNA alterations determined using a validated liquid biopsy assay suggests that prior genomic profiles derived from single time points are inadequate portrayals of the molecular alterations and clonal structures in mRCC.

This study has some limitations. First, we employed clinically available gene panel tests with selected cancer-associated genes; concurrent tissue analyses from multisite biopsies in our patients were not possible, given the nature of our real-world dataset. Second, we could not exclude the contribution of CHIP alterations in this cohort. Indeed, we conducted MONSTAR-SCREEN-2 with ctDNA molecular profiling by analyzing genomic DNA to exclude CHIP-related mutations, thus reducing the false-positive rate in ctDNA analysis [34].

In summary, this was the largest study conducted showing that ctDNA information reflects drug resistance and affects the clinical prognosis of mRCC, heralding the possibility of non-invasive detection of the mutational profile of mRCC during tumor progression. Clinical incorporation of post-progression liquid biopsy may be valuable in assessing and overcoming acquired resistance.

## Supplementary information

Supplementary Figure

Supplementary Table

Reproducibility checklist

## Data Availability

The data can be shared with investigators whose proposed use of the data has been approved by the National Cancer Center Hospital East independent review board (IRB) identified for this purpose. The data include individual participant data that underlie the results reported in this article, after deidentification (text, tables, and figures) and study protocol. Proposals to gain access to the data should be sent to kato@uro.med.osaka-u.ac.jp. Data requestors will need to sign a data access agreement.
