# Supplementary material for: Prognostic significance of circulating tumor DNA alterations in advanced renal cell carcinoma from SCRUM-Japan MONSTAR-SCREEN: a nationwide genomic profiling project
Source: Br J Cancer. 2025 May 5;133(1):111–20. doi: 10.1038/s41416-025-02985-8 (PMC12238367; doi:10.1038/s41416-025-02985-8)
Supplement: Supplementary file 1 — Supplementary Figure [file 41416_2025_2985_MOESM1_ESM.docx]

**Supplementary Data**

Contents:

Supplementary Figure S1

Supplementary Figure S2

Supplementary Figure S3

Supplementary Figure S4

Supplementary Figure S5

Supplementary Figure S6

**Supplementary Figures**

**
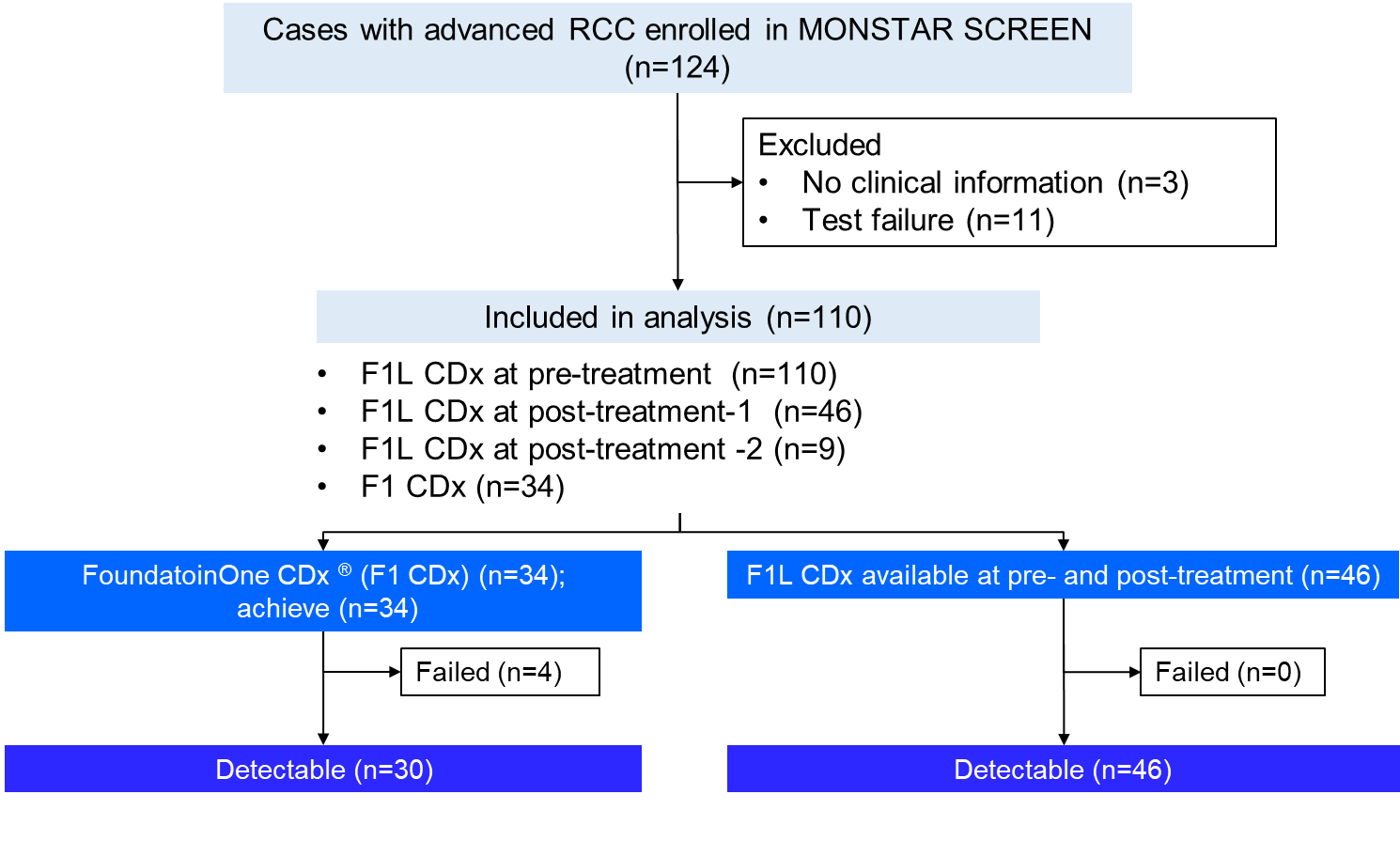
Supplementary Figure S1. Consolidated standards of reporting trial diagram in the present study.**

Post-treatment-1; Blood samples at disease progression after the first treatment. Post-treatment-2; Blood samples at disease progression after the second treatment.

**
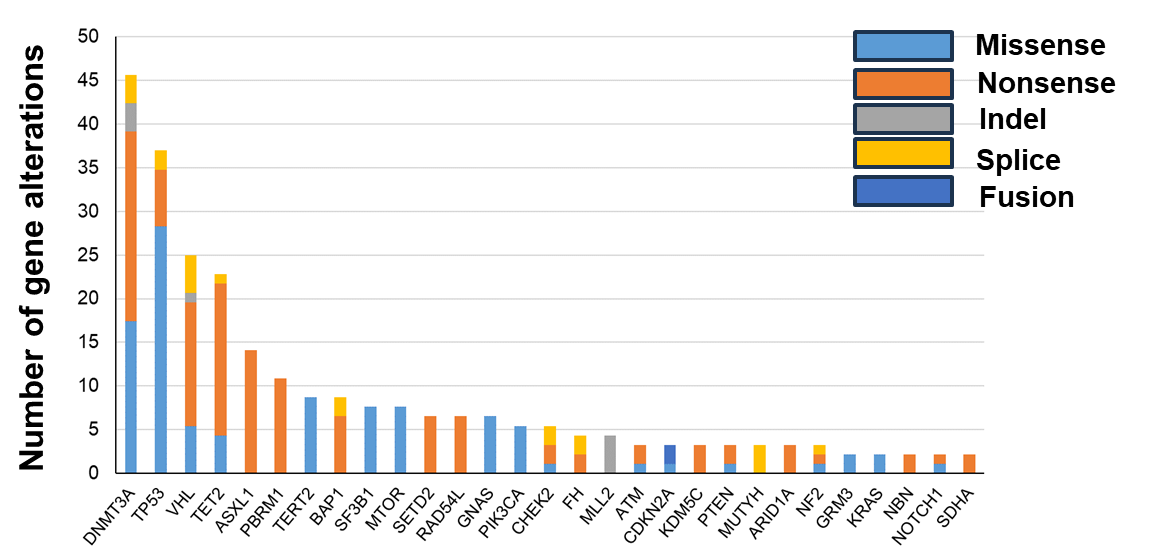
Supplementary Figure S2. The number of ctDNA gene alterations at the baseline.**

**Supplementary Figure S3. Distribution of tumour fraction at the baseline.**

**
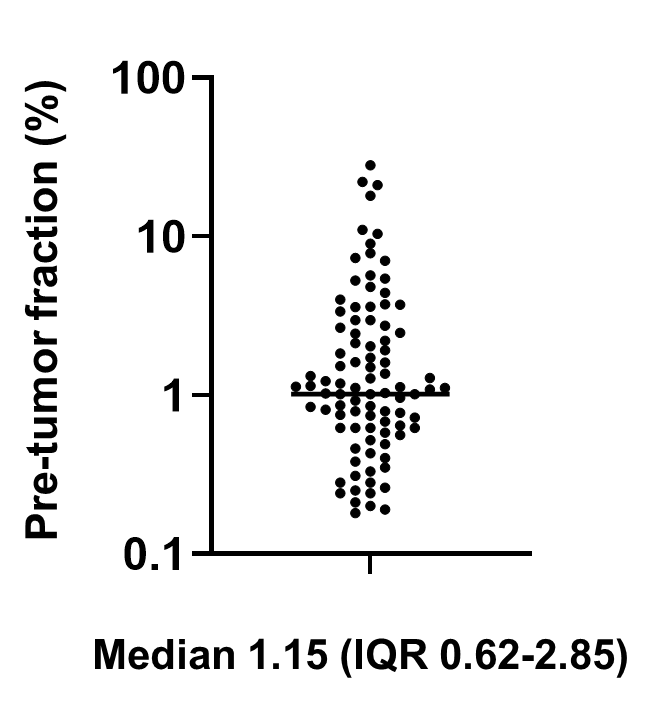
**

**
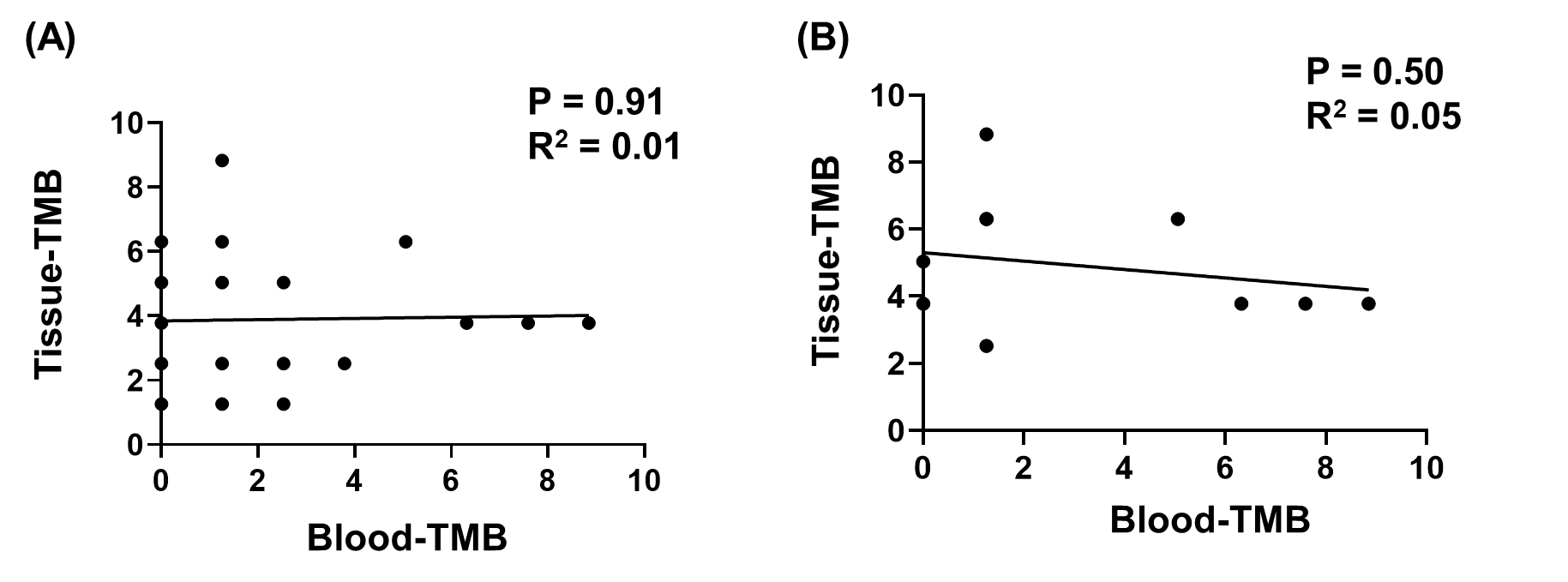
Supplementary Figure S4. Correlation between blood tumour mutation burden (TMB) and tissue-based TMB; (A) overall cohort (B) patients with tumour fraction ≥ 1%.**

**Supplementary Figure S5. Relationship between baseline gene alterations of tissue-based DNA and clinical prognosis.**


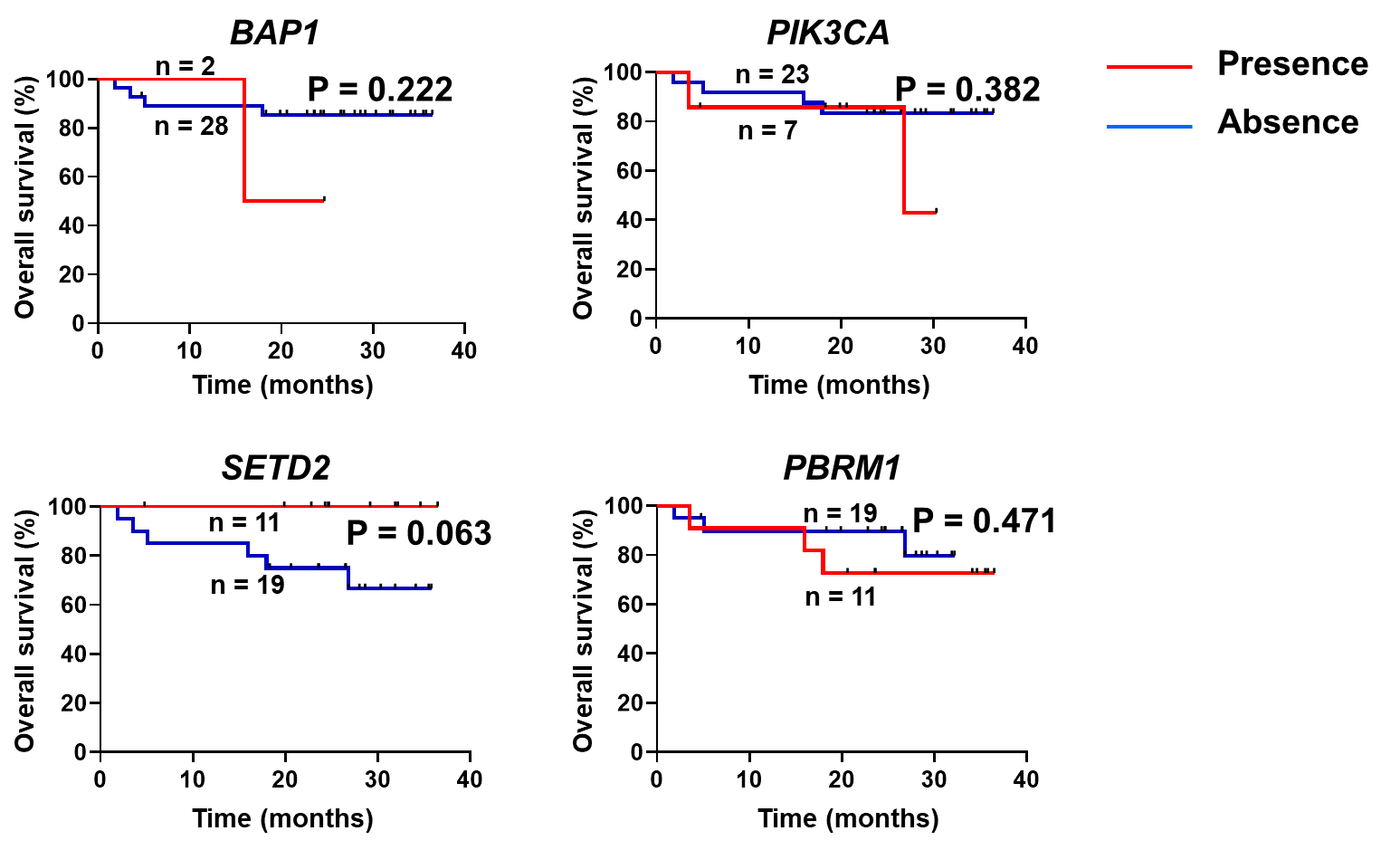
Overall survival depending on the specific mutational status of tissue-based DNA at baseline. Differences between the two groups were assessed using the log-rank test.

**Supplementary Figure S6. Therapeutic relevance of increasing or emergent ctDNA alterations.**


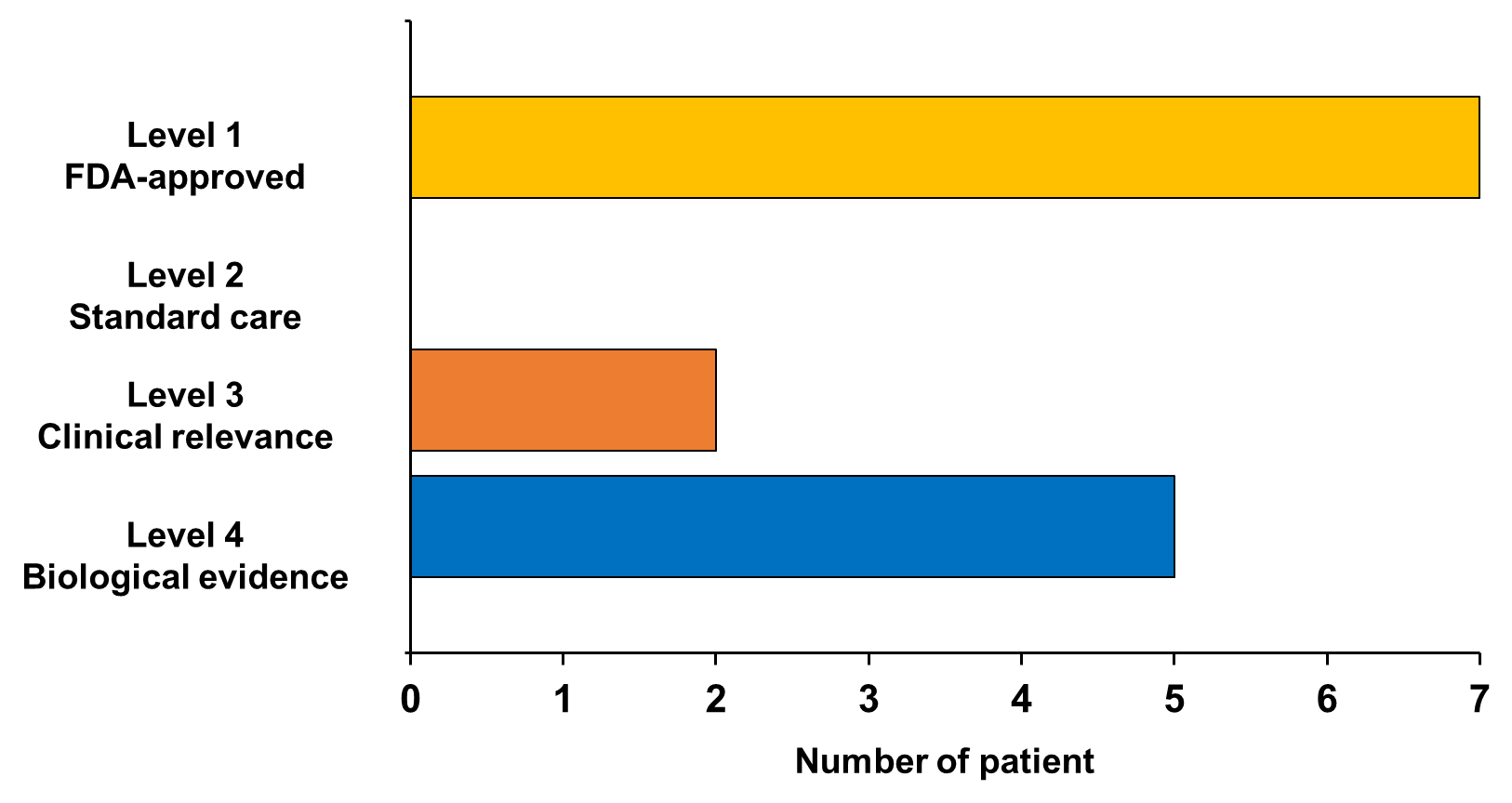
Levels of evidence used by OncoKB database.
